# Modeling of cerebellar transcranial electrical stimulation effects on hand tremor in Parkinson’s disease

**DOI:** 10.3389/fnagi.2023.1187157

**Published:** 2023-11-13

**Authors:** Soraya Rahimi, Farzad Towhidkhah, Golnaz Baghdadi, Bijan Forogh, Payam Saadat, Ghazaleh Soleimani, Seyed Amirhassan Habibi

**Affiliations:** ^1^Department of Biomedical Engineering, Amirkabir University of Technology, Tehran, Iran; ^2^Department of Neuroscience, University of Montreal, Montreal, QC, Canada; ^3^Neuromusculoskeletal Research Center, Iran University of Medical Sciences, Tehran, Iran; ^4^Mobility Impairment Research Center, Health Research Institute, Babol University of Medical Sciences, Babol, Iran; ^5^Department of Neurology, Hazrat Rasool Hospital, Iran University of Medical Sciences, Tehran, Iran

**Keywords:** computational modeling, rest tremor, transcranial alternating electrical stimulation, cerebellum, oscillators

## Abstract

**Introduction:**

Parkinson’s disease (PD) is a neurodegenerative disorder with different motor and neurocognitive symptoms. Tremor is a well-known symptom of this disease. Increasing evidence suggested that the cerebellum may substantially contribute to tremors as a clinical symptom of PD. However, the theoretical foundations behind these observations are not yet fully understood.

**Methods:**

In this study, a computational model is proposed to consider the role of the cerebellum and to show the effectiveness of cerebellar transcranial alternating current stimulation (tACS) on the rest tremor in participants with PD. The proposed model consists of the cortex, cerebellum, spinal circuit-muscular system (SC-MS), and basal ganglia blocks as the most critical parts of the brain, which are involved in generating rest tremors. The cortex, cerebellum, and SC-MS blocks were modeled using Van der Pol oscillators that interacted through synchronization procedures. Basal ganglia are considered as a regulator of the coupling weights defined between oscillators. In order to evaluate the global behavior of the model, we applied tACS on the cerebellum of fifteen PD patients for 15 min at each patient’s peak frequency of their rest tremors. A tri-axial accelerometer recorded rest tremors before, during, and after the tACS.

**Results and Discussion:**

The simulation of the model provides a suggestion for the possible role of the cerebellum on rest tremors and how cerebellar tACS can affect these tremors. Results of human experiments also showed that the online and offline effects of cerebellar tACS could lead to the reduction of rest tremors significantly by about %76 and %68, respectively. Our findings suggest that the cerebellar tACS could serve as a reliable, therapeutic technique to suppress the PD tremor.

## 1. Introduction

Parkinson’s disease (PD) is a prevalent neurodegenerative disorder. The typical symptoms include tremors, bradykinesia, akinesia, rigidity, and postural instability (Balestrino and Schapira, 2020). Inspired by Wu and Hallett (2013), patients can be divided into two groups: tremor dominant and akinesia dominant. The cerebellum, motor, sensory cortices, and diencephalic area are involved in tremor generation in tremor-dominant parkinsonian patients (Timmermann et al., 2002). Anatomical connections between the cerebellum and basal ganglia, which have been the primary clinical and research targets in the field of Parkinson’s disease (Hornykiewicz, 2006), were also discovered. These connections include a strong disynaptic projection from the cerebellum to the striatum by the thalamic route (Hoshi et al., 2005) and a disynaptic projection from the subthalamic nucleus (STN) via the pathway of the pontine nuclei to the cerebellar cortex (Bostan et al., 2010). Neuroimaging studies pointed out that the left and right cerebellum and the contralateral motor cortex are hyperactive in PD patients (Yu et al., 2007). Therefore, increasing anatomical, pathophysiological, and clinical evidence lead to recognize the involvement of the cerebellum in the clinical symptoms of PD (Yu et al., 2007; Helmich et al., 2012; Wu and Hallett, 2013; Kishore et al., 2014; Lefaivre et al., 2016).

Tremor, one of the most common PD symptoms, is described as a rhythmical, involuntary oscillatory movement of a body member (Puschmann and Wszolek, 2011). Such a tremor can reduce the quality of life from physiological and psychological aspects (Louis and Machado, 2015). Studies have shown that tremors in PD can be considered as an independent symptom resulting from less responding to dopaminergic treatments compared to rigidity and bradykinesia. Additionally, tremor progression does not happen at the same rate as other PD manifestations (Louis et al., 1999; Yu et al., 2007; Helmich et al., 2012). Invasive procedures like deep brain stimulation (DBS) can reduce tremors (Hess, 2013; Wong et al., 2019). However, researchers are seeking noninvasive approaches. Transcranial electrical stimulation (tES) and transcranial magnetic stimulation (TMS) have also been used to treat PD’s tremors (Fregni et al., 2006; Benninger et al., 2010; Brittain et al., 2013; Lefaivre et al., 2016; Marzban et al., 2017).

Previous studies tried to model rest tremors computationally based on the physiological changes in the central nervous system (Austin et al., 1965; Leondopulos and Micheli-Tzanakou, 2004; Haeri et al., 2005) and peripheral nervous system (Fukumoto, 1986; MashhadiMalek et al., 2008). Some researchers took into account only the dynamic of oscillations for modeling tremors (Beuter and Vasilakos, 1995). There is some evidence to support that rest tremors result from an involuntary running of a motor program, which finally causes rapid alternative movements (RAM) (Watson Alberts, 1972; Parker et al., 1992). Accordingly, Duval et al. (2016) suggested that the cerebellum considers rest tremors as voluntary movements and attempt to modify them in the “finger-switch-dimmer” model, a conceptual model. However, the role of cerebellum in the mechanism of generating and propagating rest tremors has not been investigated computationally.

We hypothesize that cerebellar transcranial alternating current stimulation (tACS) can support the cerebellum modulating rest tremors. This study aims to model the effectiveness of cerebellar tACS on rest tremor in PD mathematically. The proposed model is then validated by helping literature and the clinical experiment results designed and performed on a group of people with PD. To the best of our knowledge, presenting a computational model to show the contribution of the cerebellum in rest tremors is carried out for the first time in this study. In section 2, methodology and materials are presented, results are explained in section 3, followed by the discussion and conclusion in sections 4 and 5.

## 2. Materials and methods

### 2.1. Subjects

Fifteen tremor-dominant Parkinson’s patients (8 females; age 65.86 ± 12.81 years; right-handed) in the drug-off condition participated in the experiments. Demographic information is shown in Table 1. All participants signed the written informed consent form. They also completed a questionnaire concerning the possible adverse effects of tACS. Iran University of Medical Sciences (Ethics committee reference number IR.IUMS.REC.1397.1349, date 2019/02/24) approved the experiment protocol.

**Table 1:** Demographic information of PD subjects.

Factor	PD patients (*n* = 15)
Age (year)	65.86 ± 12.81
Sex (Female/male)	8/7
Dominant arm	Right
Disease duration (year)	6.5 + 2.33
Age of onset (year)	59.2 ± 9
Medication	Levodopa

### 2.2. Apparatus and experimental procedure

**Figure 1:**
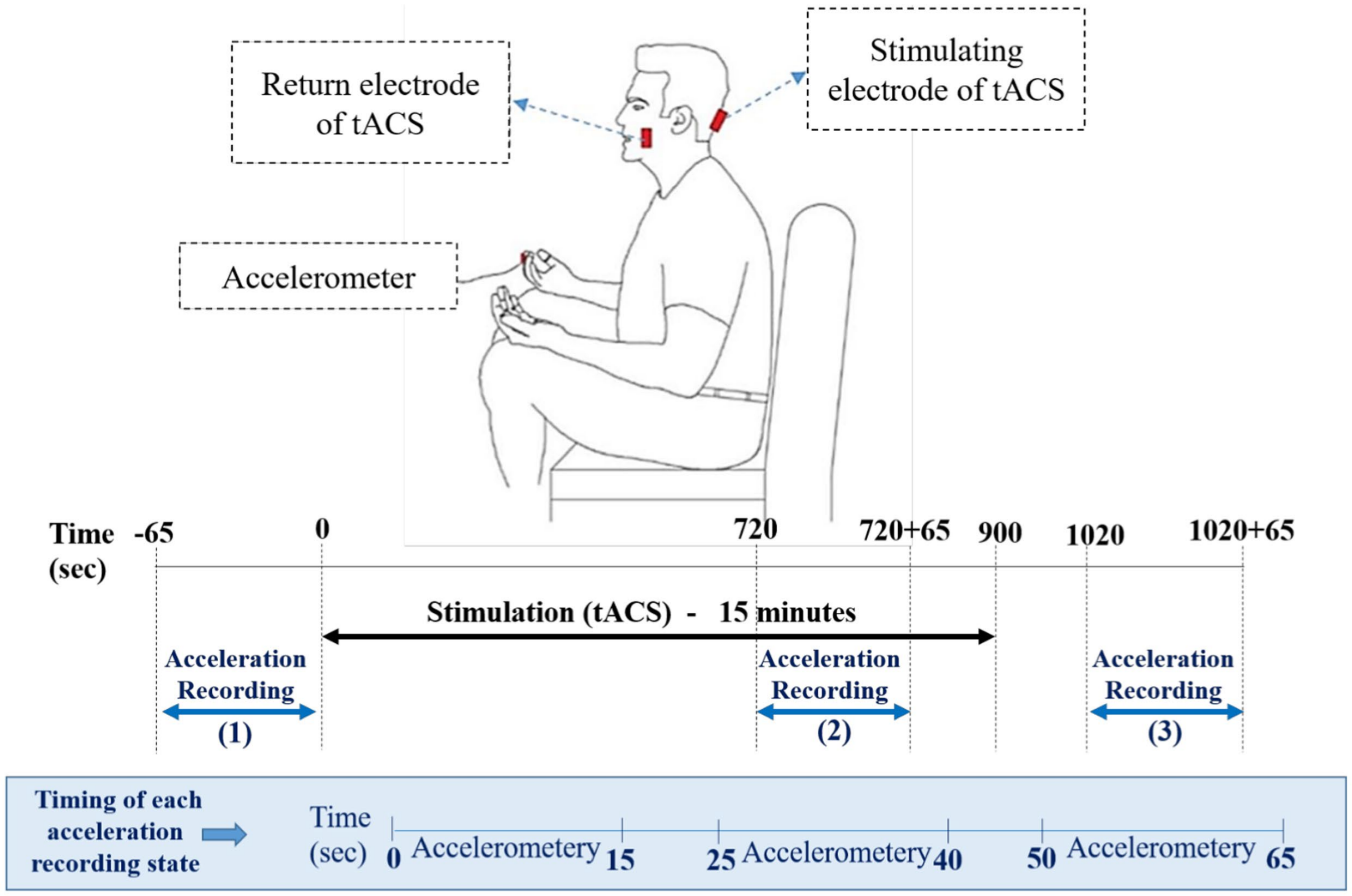
Schematic representation of hand position for the recording of rest tremors via an accelerometer. The position of active and return electrodes is shown in red over the inion and buccinator muscle. The accelerometer is placed over the index finger. Lower panels of the figure represents the timing of experiment, including acceleration recoding states and accelerometer intervals in each state.

The experiment was performed while the subjects sat on a chair comfortably with their eyes open. The participants placed their hands on their thighs during recording the rest tremor in the experiment (Figure 1). Tremor acceleration was recorded in three directions by a tri-axial accelerometer, consisting of the LIS3DH (STMicroelectronics, 2016) accelerometer sensor and the Arduino Uno board. This sensor was placed over each patient’s dominant hand index finger before starting the recording. Acceleration of rest tremors were recorded in three states, pre-stimulation, during-stimulation, and post-stimulation (Recording 1, 2, and 3). The first recording, was performed 65 s before the start of the stimulation. Stimulation (tACS) was applied for 15 min. From the 12th minute, the second recording was done for 65 s, simultaneously with the continuation of the stimulation. About 2 min after stimulation, the third 65-s recording was started. As mentioned, each recording state lasted 65 s, which included three 15-s intervals with 10-s inter recording intervals (Figure 1).

### 2.3. Stimulation protocol

**Figure 2:**
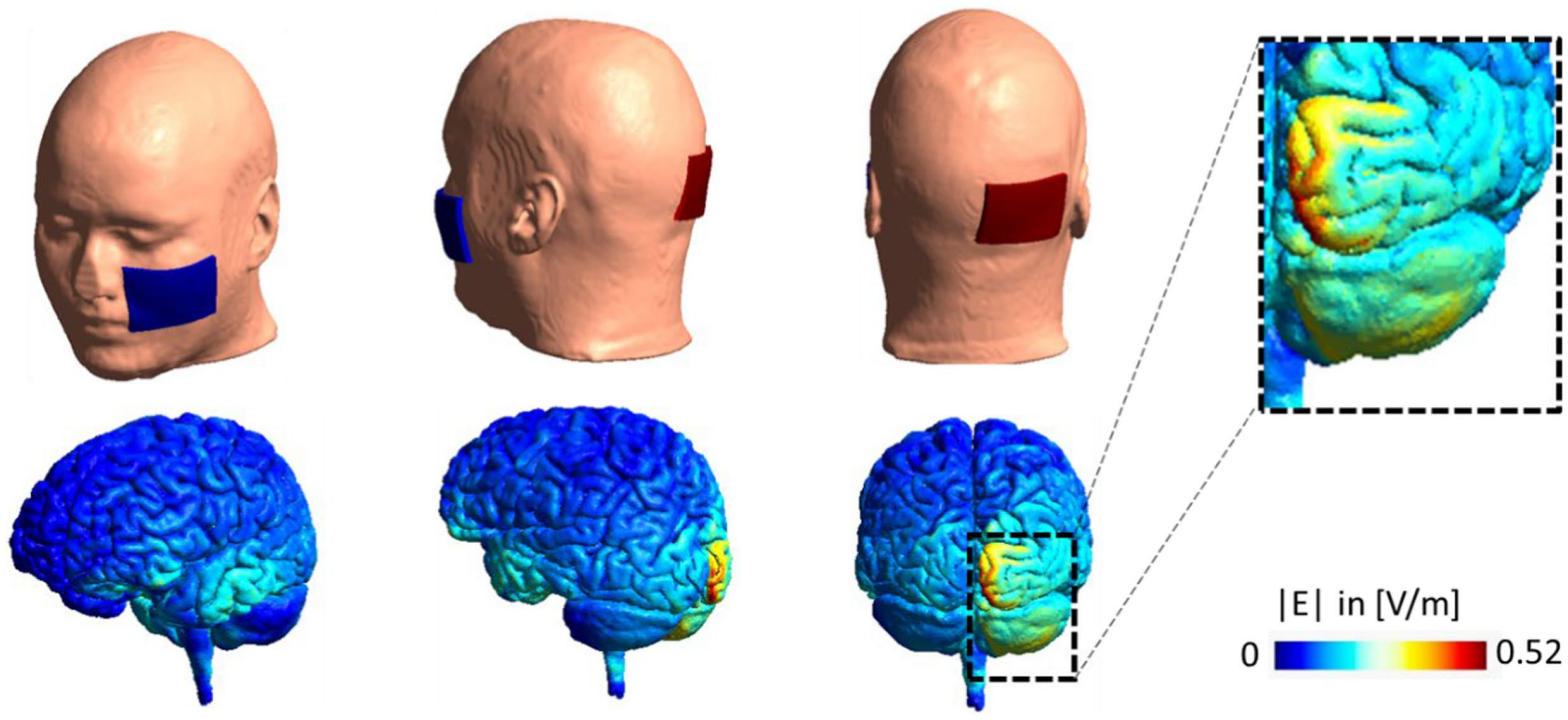
Electrode montage and electric field distribution pattern. Computational head model for a sample subject with stimulating electrode over the right cerebellum and return electrode over left buccinator muscle. A current of 2 mA is used in the stimulation. Simulation is performed in SimNIBS 3.2 using the high resolution T1-weighted image of Ernie subject in this package and EF distribution patterns are visualized over gray matter in volt per meter [V/m]. EF, electric field.

Ipsilateral cerebellar stimulation was delivered through rubber electrodes encased in saline-soaked sponges. The active electrode was positioned three centimeters lateral to the inion, and the return electrode was centered on the buccinator muscle contralateral to the recorded tremor. The applied current was a sinusoidal waveform with a peak amplitude of 2 mA for 15 min, and the electrode size was 7 × 5 cm^2^. Based on the literature (Brittain et al., 2015), tACS was applied to each patient at the peak frequency of their rest tremors, which was in the range of 4–7 Hz in the experiment. The peak frequency of participants’ rest tremors was determined through the accelerometer mentioned in the previous subsection and analyzed by using principle component analysis (PCA) in MATLAB before starting the stimulation. We simulated the electric field distribution pattern for our electrode montage in SimNIBS 3.2. The simulation results are represented in Figure 2.

### 2.4. Analyzing experimental data

In preprocessing, data from the accelerometer was smoothed by performing a cubic spline interpolation. Then, the average resultant acceleration (RA), approximate entropy (ApEn), and the peak frequency were extracted as features. The resultant acceleration of rest tremor was calculated as follows:


(1) $$RA=\sqrt{x^{2}+y^{2}+z^{2}}$$

*x*, *y*, and *z* are axis directions. The ApEn method quantifies the complexity and synchrony of time series. This ApEn value was computed by Eqns. 2–6 (Pincus et al., 1991; Gil et al., 2010). Fast Fourier transform (FFT) helped us determine the amplitude and frequency of the peak frequency in the frequency domain.


(2) $$\mathrm{ApEn}=\varphi^{m}\left(r\right)-\varphi^{m+1}\left(r\right)$$

(3) $$\varphi^{m}\left(r\right)={\left(N-m+1\right)}^{-1}\overset{N-m+1}{\underset{i=1}{\sum }}\log\left(C_{i}^{m}\left(r\right)\right)$$

(4) $$\begin{array}{l} C_{i}^{m}\left(r\right)=\left(\mathrm{number}\;\mathrm{of}\;x\left(j\right)\;\mathrm{such}\;\mathrm{that}\;d\left[x\left(i\right).x\left(j\right)\right]\right)\\ \;/\left(N-m+1\right) \end{array}$$

(5) $$d\left[x.x^{*}\right]=\max_{a}|u\left(a\right)-u^{*}\left(a\right)|$$

(6) $$x\left(i\right)=\left[u\left(i\right).u\left(i+1\right).\ldots .u\left(N-m+1\right)\right]$$

There are *N* raw data in a time series of data $u\left(1\right).u\left(2\right).\ldots .u\left(N\right)$ . *m*, an integer, represents the length of the sequence to be compared. *r*, a positive real number, measures a filtering level for matching similarity between two segments.

### 2.5. Modeling

**Figure 3:**
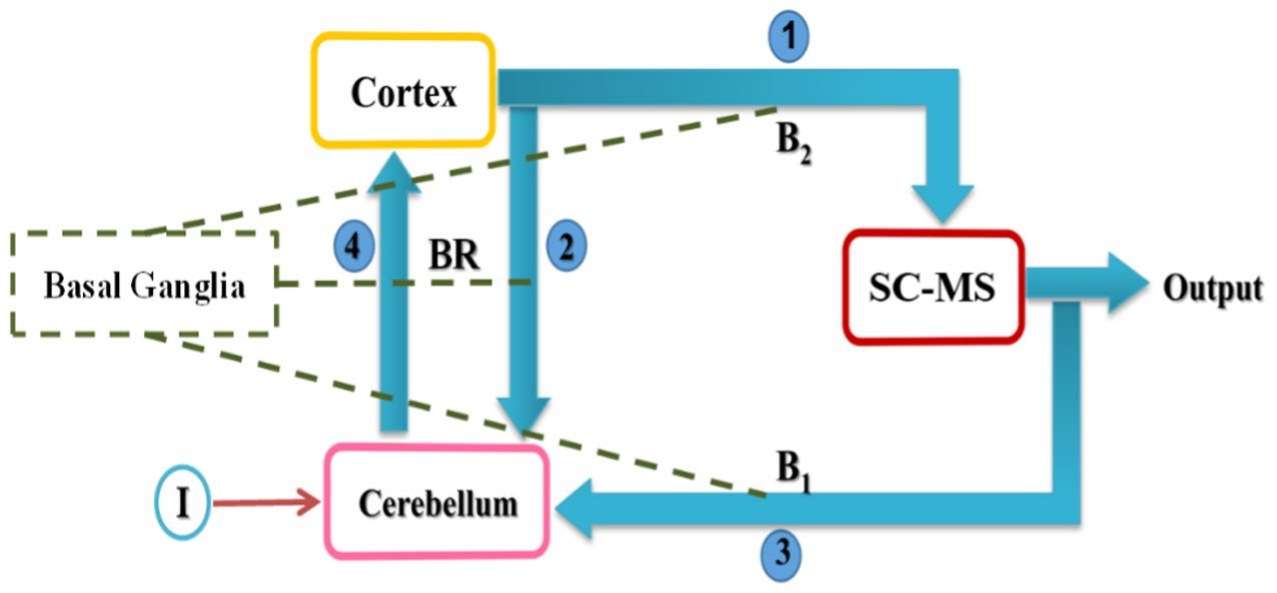
Conceptual model of rest tremor in PD. SC-MS block is a set of spinal circuits and muscular systems. Arrows represent the primary connections among the cortex, cerebellum, and SC-MS, while dashed lines indicate the effect of basal ganglia on neurotransmitters. I and BR denote input (tACS) and mutual relationship between cortex and cerebellum, respectively.

We proposed a computational model to show the effectiveness of the cerebellar tACS on rest tremor based on biological findings. Studies have shown that there can be a loop between the cortex, cerebellum, basal ganglia and spinal circuitry and muscle systems which are involved in tremor modulation (Hoshi et al., 2005; Yu et al., 2007; Bostan et al., 2010; Wu and Hallett, 2013). Additionally, tACS can reduce tremors by altering neuronal oscillations (Brittain et al., 2013, 2015). Figure 3 represents the block diagram of the proposed model proposed. This model consists of four main components: Cortex, Cerebellum, spinal circuits and muscular systems (SC-MS), and Basal Ganglia.

Due to the prominent role in dopamine production (Hornykiewicz, 2006), basal ganglia are considered a regulator of the coupling weights defined between oscillators.

In Figure 3, cortical oscillations are sent to the SC-MS block with connection 1 (Lemon, 2008), and a copy of that is sent to the cerebellum block with connection 2. Connection 1 plays the role of efferent signals in the brain. Then, the SC-MS block gives the feedback of the executed movement to the cerebellum block through connection 3 (Ramnani, 2006). Connection 3 is equivalent to afferents to the cerebellum. The cerebellum compares the desired output with the actual output and sends a message to the cortex via connection 4 (Ramnani, 2006; Flanders, 2009).

Since neuronal sets can be considered as oscillators (Stiefel and Ermentrout, 2016), blocks in Figure 3 are modeled by oscillators that can interact with each other. These interactions take place with the process of synchronization or desynchronization behaviors observed during voluntary movements and rest tremor (Balanov et al., 2009). Oscillatory units are designed to exhibit synchronization and desynchronization behaviors. When the interaction of two systems leads to adapt their time scales, they synchronize with each other (Balanov et al., 2009). Adjustment and regulation of the time scale result from mutual or forced synchronization. In the proposed model, it causes units to oscillate at similar frequencies, and the phase is gradually aligned between them and their natural dynamics may suppress as well. Desynchronization happens against the synchronization, which means a variable phase/frequency difference between the units’ oscillations. Regulation of coupling weights can play essential roles in happening frequency/ phase locking.

We have modeled the cortex, cerebellum, and SC-MS with Van der Pol oscillators, which have been used to model tremors in previous studies (Austin et al., 1965; Timmer et al., 2000; Harikrishna et al., 2016) and are well-known in modeling the biological systems. Van der Pol oscillator simplifies the model resulting from not considering the neuronal details and chemical reactions (Baghdadi et al., 2019). Furthermore, the results of the simulation could satisfy the primary goal of the model.

Equation 7 shows the mathematical representation of a Van der Pol oscillator.


(7) $$\overset{¨}{Y}-\left(\lambda -Y^{2}\right)\dot{Y}+p^{2}Y=0$$

The output of the oscillator is represented by the *Y* value. The frequency and the amplitude of oscillations are, respectively, determined by the value of *p* and λ . In this equation, λ is the bifurcation parameter (i.e., the changes of this parameter can cause considerable alternation of the output pattern), in that λ≤0 leads to the suppression of oscillations and when λ is greater than one, the oscillations have multiple harmonics.

The bifurcation parameters of Van der Pol oscillators should be 0<λ≪1 to have a single frequency of oscillation that is determined by *p* value (Baghdadi et al., 2019). As a result, the model becomes simpler. Thus, in all simulations, $\lambda_{co}=\lambda_{ce}=\lambda_{p}=0.3$ . It is worth to be noted that other values between zero and one lead to the same pattern of results obtained and reported in this study.

The value of *p* for each block has been determined based on previous studies that provide an estimation of the frequency of these units’ activities. The frequency ranges of cortex, cerebellum and the SC-MS activities (three units of the proposed model) were measured by MEG and EEG (Hari, 2006), microelectrode recordings (Groth and Sahin, 2015), accelerometer and surface EMG techniques (Bain, 2002; Dirkx et al., 2018) respectively. These results are indicated in Table 2 for healthy subjects.

**Table 2:** Frequency range of each units’ activities.

Blocks	Frequency (Hz)
Cortex (Hari, 2006)	10–20
Cerebellum (Groth and Sahin, 2015)	4–25
SC-MS (Bain, 2002, Dirkx et al., 2018)	7–12

Accordingly, the *p* values (intrinsic frequencies) of the units of the proposed model were set as follows in Equations 8–10, which are the main equations of the proposed model:


$$p_{co}=15\mathrm{Hz}.p_{ce}=20\mathrm{Hz}.p_{s}=10\mathrm{Hz}\text{.}$$

We considered three Van der Pol oscillators, which represent the loop involved in executing voluntary movements, including the cortex, cerebellum, SC-MS.


(8) $${\overset{¨}{Y}}_{co}-\left(\lambda_{co}-Y_{co}^{2}\right){\dot{Y}}_{co}+p_{co}^{2}Y_{co}=BR\left(Y_{ce}-Y_{co}\right)$$

(9) $${\overset{¨}{Y}}_{ce}-\left(\lambda_{ce}-Y_{ce}^{2}\right){\dot{Y}}_{ce}+p_{ce}^{2}Y_{ce}=BR\left(Y_{co}-Y_{ce}\right)+B_{1}Y_{p}+I$$

(10) $${\overset{¨}{Y}}_{s}-\left(\lambda_{s}-Y_{s}^{2}\right){\dot{Y}}_{s}+p_{s}^{2}Y_{s}=B_{2}Y_{co}$$

The subscripts *co*, *ce*, and p, respectively, represent the cortex, cerebellum, and SC-MS. The term I denotes an input to the cerebellum, which is tACS in the proposed model. The coupling weights between units (*BR* and *B_i_*) represent synapses in which neurotransmitters influence the performance. In this study, the most notable neurotransmitter is dopamine. According to the synchronization between cortex and cerebellum (Timmermann et al., 2002), we considered that they interact with each other through mutual synchronization, which leads to adding the new term $BR\left(Y_{co}-Y_{ce}\right)$ to Eqns. 8 and 9. It shows the effect of cortex on the cerebellum and vice versa. $B_{1}$ represents the influence of sensory feedback on the performance of the cerebellum, and $B_{2}$ determines the influence of the cortex on the SC-MS. These coupling weights are used as control variables to represent the healthy and PD states in the model.

As mentioned before, the effect of tACS, as an external oscillator, can be inserted into the model, as shown in Eqn. 9 by the symbol *I*. This external oscillator (tACS) is modeled by Eqn. 11. Few studies investigated the offline-effect of tACS. They suggested that the stimulation duration (Strüber et al., 2015) and the endogenous power of oscillations (Neuling et al., 2013) can impact the offline-effect of tACS. In this study, an exponential term is added to Eqn. 11, and the result is shown in Eqn. 12 to simulate the decreasing effect of electrical current on neurons after removing the electrodes from the skull.


(11) I=Asinwt

(12) $$I={Ae}^{-bt}\sin\mathrm{wt}$$

**Figure 4:**
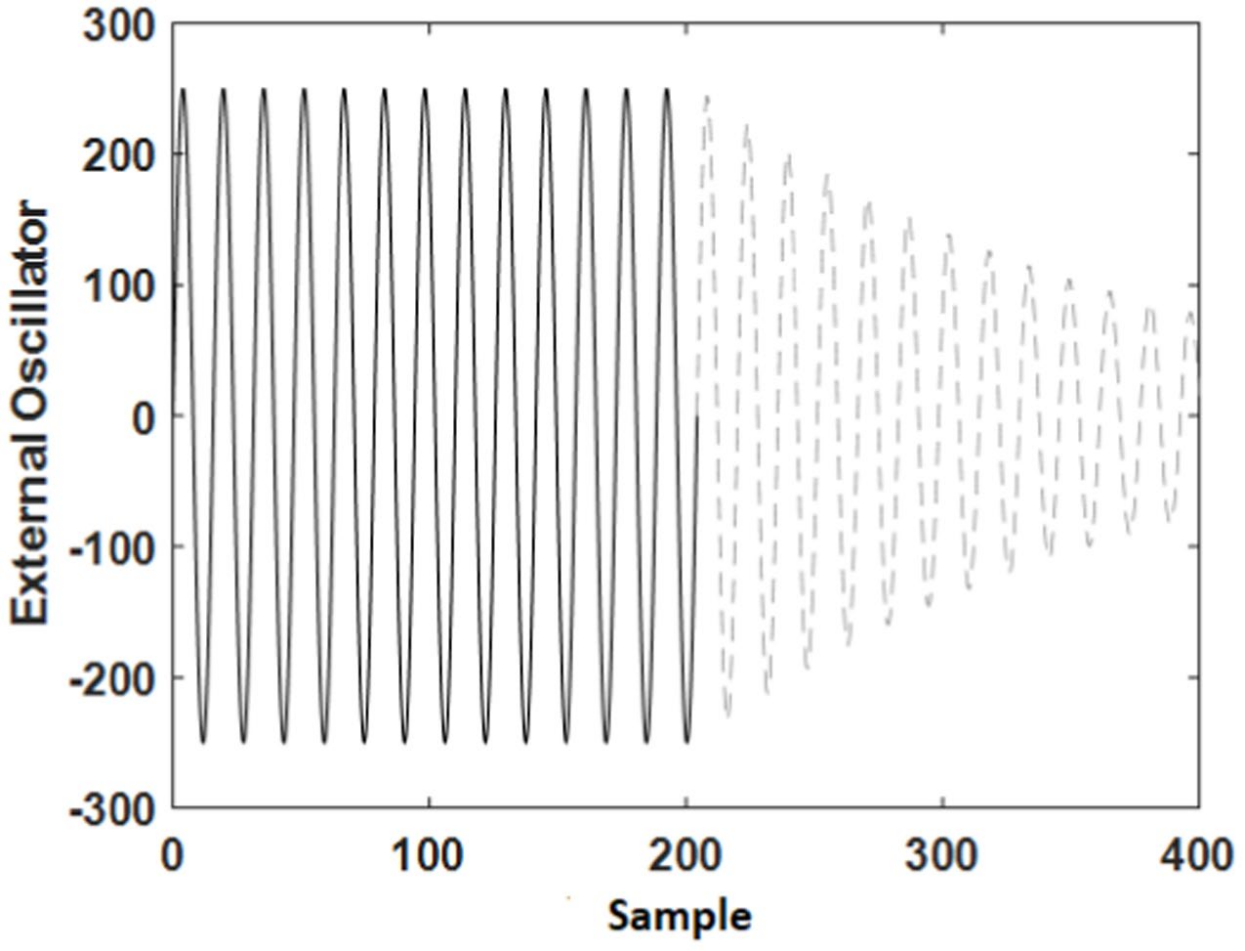
Simulation of the external oscillator (tACS) for online-effect (solid black line) and offline-effect (dash gray line).

Figure 4 is an example to display the shape of external input simulation considering the conditions described by Eqns. 11 and 12. This input is inserted to the cerebellum block to consider online- and offline-effects of tACS on the model. A sinusoidal wave is presentative of online effects (during the stimulation), which is shown by solid line. In other words, as long as sinusoidal wave is inserted to the model, it behaves like during stimulation in the experiments. Subsequently, we assumed offline effects such as an exponential term which the its amplitude decrease in time (the dash line color in Figure 4).

Proposed model is used to simulate healthy and PD states. Decrease of coupling weights get model from healthy state into PD state because these coupling weights are a representation of the dopamine level, which is lower in people with PD. Considered blocks involved in the rest tremor generation may desynchronize with each other in a healthy state. When synchronization occurs, the cortex and cerebellum blocks become hyperactive, and the behavior of the model is like the PD state (Timmermann et al., 2002; Yu et al., 2007). According to these changes, coupling weights are adjusted between 30 to 40 in the healthy state and 1 to 10 in the PD state. The limited range of coupling weights is in agreement with the fact that there is a specific limit of neurotransmitters in healthy subjects. For instance, the increment of the dopamine level over the normal range can cause some diseases or disorders (Hague et al., 2005).

### 2.6. Statistical analysis

We used Kolmogorov–Smirnov test to determine if the distributions of two samples come from a normal distribution. After confirming the non-normality of the data, the Wilcoxon signed-rank test was used for statistical analysis to assess whether the mean of signals differs during and post-stimulation compared to the pre-stimulation. The null hypothesis for this test was that the means of two samples are equal. In this study, a *p*-value of less than 0.05 was considered statistically significant. All the processing and analyses were performed in MATLAB 2018b software.

## 3. Results

### 3.1. Model simulation results

**Figure 5:**
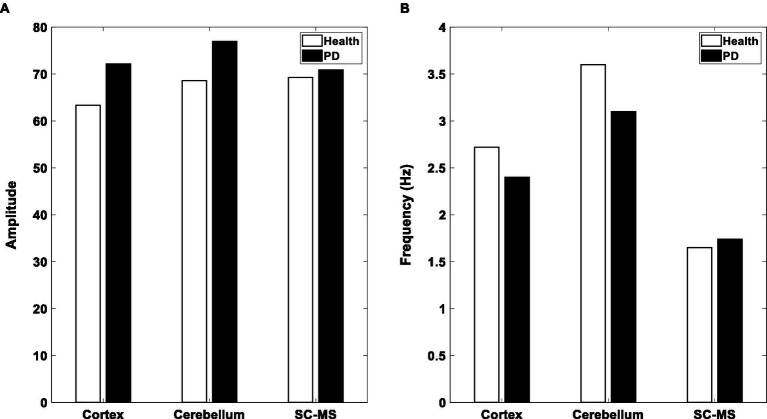
Output of three blocks in the Model. **(A)** Amplitude of the considered oscillators’ activities in the proposed model in healthy (white bar) and PD (black bar) states. **(B)** The peak frequency of the oscillators in the healthy and PD states. SC-MS, spinal circuit and muscular system.

As it has been mentioned in the Method part, coupling weights are the control parameters in the proposed model. That is, the changes of these parameters, which are a representation of the dopamine level, can alter the output behavior from healthy to PD. In this section, the results of the model simulation are presented by altering the coupling weights, and also the effect of tACS input on the model output, which is a representation of the tremors. The unit’s activities increase by decreasing the coupling weights, as shown in Figure 5A. Indeed, after a while, the phase of the three oscillators gradually become similar to each other. Then, as a result of the between-unit synchronization, the activation of units begins to increase. Results agree with this claim that the cerebellum and cortex are hyperactive in the PD (Wu and Hallett, 2005; Wu and Hallett, 2013). The model results are illustrated in Figure 5B in frequency domain. This figure shows the oscillators’ peak frequencies declined by changing the model mode from the healthy state to the PD state. These results are consistent with decrease of frequency range in PD as mentioned in DeLong and Wichmann (2007).

The simulation results show a significant reduction of ApEn from a healthy state to the PD state, from 0.55 to 0.27. Gil et al. (2010) also observed a decline in ApEn in PD patients compared with normal subjects.

**Figure 6:**
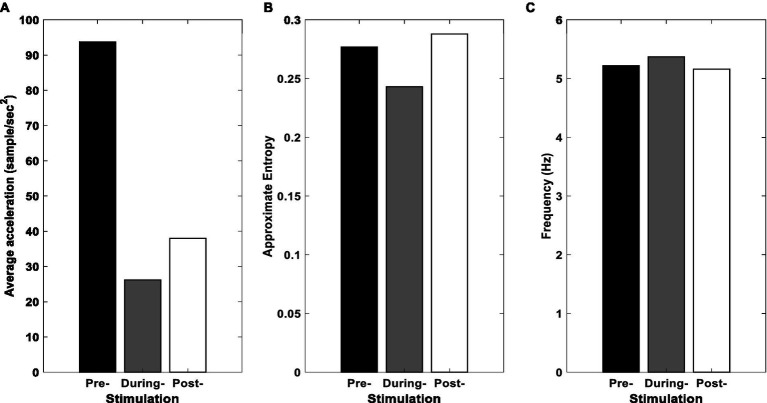
Results for simulation of SC-MS block. **(A)** Average acceleration, **(B)** ApEn, and **(C)** peak frequency of the proposed model’s SC-MS block pre-, during, and post-stimulation.

Figure 6A illustrates the average amplitude of oscillations of the SC-MS block for healthy and PD state. According to Figure 6A, the average acceleration experienced a remarkable reduction during the stimulation. After applying tACS, a slight increase can be seen in the average acceleration. Figure 6B shows that tACS leads to a reduction in ApEn during the stimulation and a rise in the post-stimulation on the ApEn. When tACS was being delivered to the cerebellum, PD patients experienced the smallest hand tremor. The frequency of a peak in the frequency domain remains steady during stimulation and post-stimulation, as seen in Figure 6C.

### 3.2. Experimental results

**Figure 7:**
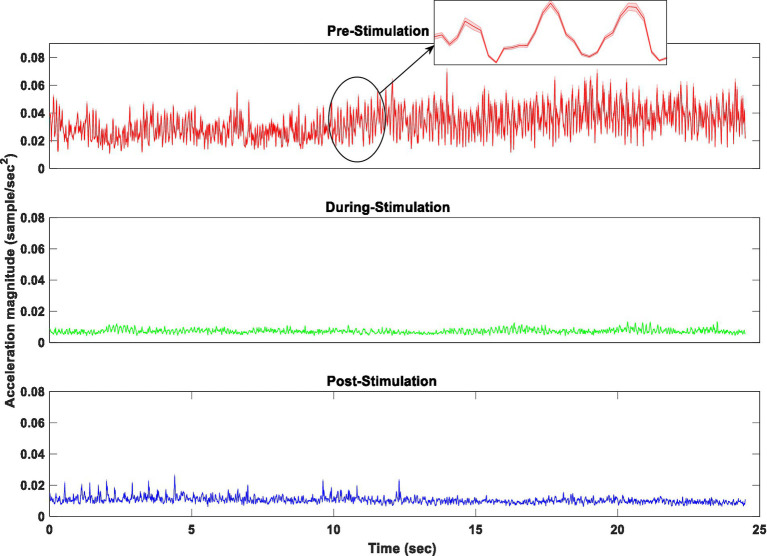
The average acceleration magnitude of fifteen subjects in three conditions of pre, during, and post-stimulation.

Figure 7 displays the average resultant acceleration (RA) of rest tremors of fifteen subjects, recorded from participants in three conditions of pre-, during-, and post-stimulation.

**Figure 8:**
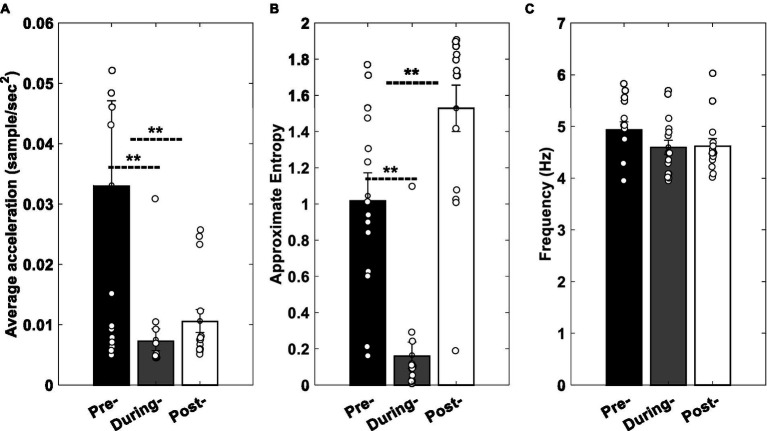
Experimental results. **(A)** Average acceleration, **(B)** ApEn, and **(C)** peak frequency of clinical data in pre-, during, and post-stimulation conditions. Error bars indicate standard error of mean values, and stars show the statistically significant differences between conditions. Circles show the values related to each participant.

Statistical analysis of the effect of the stimulation (pre-, during-, and post-stimulation) on average, approximate entropy (ApEn), and frequency of the tremors’ RA revealed that the average RA of the rest tremors decreased significantly by about 76% (*p* = 0.0052) during the stimulation and 68% (*p* = 0.0353) after the stimulation (Figure 8A). The average ApEn declined approximately 84% (*p* = 0.0006) during the stimulation, while it increased by 50% (*p* = 0.041) in the post-stimulation condition (Figure 8B). The average of peak frequency dropped by 9.73% (*p* = 0.107) and 4.68% (*p* = 0.094) during and after the stimulation, respectively (Figure 8C).

Figure 6 shows the changes pattern of the model’s output while the tACS input (*I* in Eqn. 9 described in Modeling section) has a zero amplitude (pre-stimulation), a fixed amplitude greater than zero (during stimulation), and a gradually declining amplitude (post-stimulation). Similarly, Figure 8 demonstrates the changes pattern of signals recorded from participants’ tremors in three mentioned conditions (pre-, duration, and post-stimulations). A comparison of Figures 6A,B and Figures 8A,B show that the behavior of the model simulation and experimental results are consistent qualitatively. That is, in both, the values of the average acceleration and approximate entropy decrease by applying the stimulation input, while increase in post-stimulation condition. This increment in the approximate entropy is greater than the average acceleration.

### 3.3. Further investigations of the model behavior

**Figure 9:**
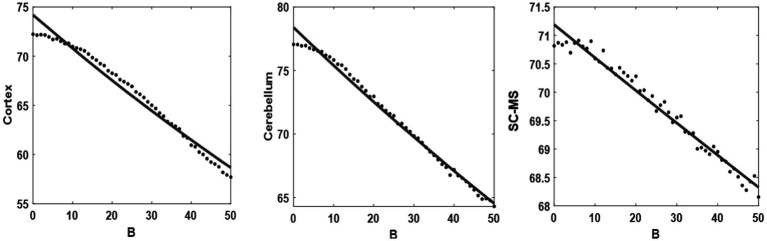
Regression model. Fitting the exponential function (solid line) onto the proposed model output (black points) with different values of coupling weights (B) (from right to left: cortex, cerebellum, and SC-MS blocks).

In previous section, it is showed that the model simulations and experimental results are in agreement qualitatively. The model can also reveal the possible relationship between the value of coupling weights (B, a representation of the dopamine level) and the average acceleration of the output (a representation of the average rest tremor acceleration). Dotted lines in Figure 9 show the average acceleration value of each model units’ output while changing the coupling weights (BR in the model’s equations, describe in Modeling section). In order to find the mentioned relationship, we tried to find the best regression model that fits to the calculated data (dotted lines). Exponential functions ( ${ae}^{bB}$ ) were the best models with lower error of fitting (solid lines in Figure 9). The value of a and b for each units of the model reported in Table 3. The slope of the fitted lines (solid lines in Figure 9, or exponential functions in Table 3) shows how much the activity of each unit is sensitive to the changes of B value (dopamine level). This sensitivity can be calculated by the derivative of fitted exponential functions with respect to the variable B. As described in Modeling section, coupling weights are adjusted between 30 and 40 in the healthy state and 1 to 10 in the PD state. Therefore, the values of the mentioned derivative in the range of 30 < B < 40, and 1 < B < 10, suggest the sensitivity of units to the changes of B value (dopamine level) in the healthy and PD states, respectively. Results of sensitivity in Table 3 suggest that people with PD are less sensitive to the changes of B values (i.e., the absolute values of sensitivity in PD state is lower than the healthy state).

**Table 3:** The function and sensitivity value in the range of healthy and PD states.

Blocks	Function	Sensitivity	
	$f\left(B\right)=ae^{bB}$	Healthy	PD
Cortex	*a* = 74.58 *b* = −0.0049	−0.3849	−0.1356
Cerebellum	*a* = 71.24 *b* = −0.00085	−0.3083	−0.1303
SC-MS	*a* = 78.43 *b* = −0.0038	−0.0655	−0.0132

## 4. Discussion

We proposed a computational model that can show the effectiveness of the cerebellar tACS on rest tremor based on biological findings. An experiment was carried out on PD patients to evaluate the global behavior of the model. Experimental results revealed that the online and offline effects of tACS on the rest tremor’s acceleration were about 76 and 68%, respectively (Figure 8A). This influence is greater than the effects of TMS (Lefaivre et al., 2016), tDCS (Fregni et al., 2006), and M1-tACS (Brittain et al., 2013) on the PD tremor.

In this study, tACS as an external oscillator with a sufficiently close frequency to the cerebellum’s frequency, as an internal oscillator, was applied to the cerebellum. The literature states that the frequency of tremors is approximately equal to the cerebellum frequency of activities (Brittain et al., 2013, 2015). By reducing coupling weights and going into the PD state, three oscillators’ phases gradually synchronize with the external forcing of tACS. As a result of the synchronization between oscillators, units’ activation begins to increase (Grabska-Barwińska and Żygierewicz, 2006). Therefore, hyperactivity occurs in the cortex, cerebellum, and SC-MS blocks in the PD state, shown in Figure 5A.

In a comparative study between essential and Parkinson’s tremors, Brittain et al. investigated how multiple neural oscillators contribute to PD patients’ frequency tolerance profiles. Based on the results, the broad frequency tolerances observed in the PD cohort can be explained by the superposition of uncoupled oscillators that do not have a strong tendency to oscillate at a specific frequency. As a result, we can conclude that tremors are confined to certain resonance margins, and the peripheral manifestation of tremors diminishes as the central drive exceeds these margins. They also proposed the intriguing possibility that external stimulation, such as tACS, could potentially drive tremors outside their tolerance zone, leading to a reduction in tremor amplitude and associated disability (Brittain et al., 2015). In the current study, we aim to modulate the abnormal neural oscillations that cause tremors. In order to reduce the severity of tremors, tACS may entrain or desynchronize these oscillations. There is, however, a possibility that the optimal stimulation frequency will differ from individual to individual. Therefore, in our experiment tACS was applied to each patient at their peak rest tremor frequency, which was 4–7 Hz.

Changing the stimulus amplitude in tACS affects the strength of the electrical current delivered to the brain. An increase in electrode amplitude can be associated with more substantial neural modulation, while a decrease in electrode amplitude may produce weaker effects. A balance must be struck between efficacy and safety when determining amplitude. In contrast, higher amplitudes outside the range may cause discomfort or adverse effects, while lower amplitudes may be less effective.

The complexity of tremors is determined by the combination of mechanical and neural effects (Morrison and Newell, 2000). Given the synchronization of the considered oscillators in the PD state, there are more regular oscillations in the rest tremors resulting in the lower ApEn in the PD state than the healthy state. Furthermore, the literature indicated that the effects of aging and disease might be associated with a reduction in system complexity (Morrison and Newell, 2000; Gil et al., 2010). Thus, an increase in ApEn can be indicative of an improvement in patients with PD. Offline-effects in Figure 6B support this hypothesis compared to the pre-stimulation.

As shown in Figures 6C, 8C, slight changes in frequency can be due to our choice of the stimulation frequency, which is around equal to the peak frequency of rest tremor in the pre-stimulation (Brittain et al., 2015).

The exponential pattern of the model output with the different coupling weights (Figure 9) is consistent with the aforementioned physiology of neurotransmitters. The negative slope in Table 3 also shows the downward trend in the neuronal activity with increasing neurotransmitters. The greater value of the sensitivity of the healthy state than the PD state represents that the velocity in a healthy state is higher than the PD state. In addition, the speed of synchronization between the oscillators increases by rising coupling weights resulting in the reaction time decline. This result offers confirming evidence for the fact that the reaction time in PD patients decrease compared with healthy subjects (Hu et al., 2019). Moreover, the negative acceleration (second derivative) is observed by increasing coupling weights regarding the sign of a and b coefficients in Table 3, which can be representative of less tremor. Results of sensitivity reported in Table 3 show that people with PD are less sensitive to the changes of B values (dopamine level). That is, they may need higher level of dopamine to have the same pattern of healthy activities. In other words, the model predicts that people with PD lose their sensitivity to the changes of the dopamine level. However, this prediction needs clinical investigations in future studies.

Although the exact mechanism of tACS on the cerebellum is not fully understood, and research in this area is ongoing, there are a few mechanisms that may explain the effects of tACS on cerebellar function. A possible mechanism for tACS on the cerebellum involves neural oscillation entrainment. In targeted brain regions, electrical stimulation at specific frequencies can synchronize or entrain oscillatory activity. It may be possible to modulate motor control processes by utilizing tACS in the cerebellum by modulating oscillatory activity in the cerebellar cortex and deep cerebellar nuclei. Another potential mechanism is the modulation of neuronal excitability. Several studies have shown that electrical stimulation affects neuron excitability in the stimulated area. In the cerebellum, tACS can alter the neuronal excitability, which may affect the overall functioning of motor control circuits. Considering the mechanism of the tACS effects on the cerebellum, it is also important to note that a variety of parameters, including stimulation frequency, intensity, and duration, can influence tACS effects on brain regions.

The proposed computational model brings new insight into the cerebellum’s role in suppressing rest tremor using tACS. However, the experiment was conducted with a limited number of participants and lack of sham group in this work. Thus, it is suggested that further investigations could be performed by increasing the number of participants, integrating with functional neuroimaging, computational head models for future works.

In conclusion, this model can make it possible to predict the system’s output behavior with a different value of neurotransmitters. Furthermore, the online- and offline-effects of cerebellar tACS on rest tremors were statistically significant, proving that the cerebellum is one of the involved brain regions in rest tremors. Moreover, our findings show that not only the cerebellum can be a target in PD patients but also cerebellar tACS has a great potential to be a novel therapy to control rest tremor. It may serve as a reliable technique to re-establish the brain oscillations’ physiological balance, particularly in PD patients.

## Data availability statement

The raw data supporting the conclusions of this article will be made available by the authors, without undue reservation.

## Ethics statement

The studies involving human participants were reviewed and approved by the Iran University of Medical Sciences (IR.IUMS.REC.1397.1349). The patients/participants provided their written informed consent to participate in this study.

## Author contributions

SR and FT designed the research study. SR, BF, and SH conducted the experiments. GS provided valuable help and advice on the stimulation protocol. SR and GB analyzed the data and wrote the manuscript, contributed to the editorial changes in the manuscript. PS assisted with participant recruitment. All authors approved the final version for submission.

## References

[ref1] Austin G. Hayward W. Tsai C. Kuykendall A. (1965). Parkinsonian tremor: some aspects of an experimental model and its solution. Stereotact. Funct. Neurosurg. 26, 389–403. doi: 10.1159/000104056, PMID: 5329843

[ref2] Baghdadi G. Towhidkhah F. Rostami R. (2019). A mathematical model of the interaction between bottom-up and top-down attention controllers in response to a target and a distractor in human beings. Cogn. Syst. Res. 58, 234–252. doi: 10.1016/j.cogsys.2019.07.007

[ref3] Bain P. G. (2002). The management of tremor. J. Neurol. Neurosurg. Psychiatry 72, I3–I9. doi: 10.1136/jnnp.72.suppl_1.i3 PMID: 11870197 PMC1765579

[ref4] Balanov A. Janson N. Postnov D. Sosnovtseva O. (2009). Synchronization: from simple to complex. Berlin: Springer.

[ref5] Balestrino R. Schapira A. H. V. (2020). Parkinson disease. Eur. J. Neurol. 27, 27–42. doi: 10.1111/ene.14108 31631455

[ref6] Benninger D. H. Lomarev M. Lopez G. Wassermann E. M. Li X. Considine E.. (2010). Transcranial direct current stimulation for the treatment of Parkinson's disease. J. Neurol. Neurosurg. Psychiatry 81, 1105–1111. doi: 10.1136/jnnp.2009.202556 20870863 PMC4162743

[ref7] Beuter A. Vasilakos K. (1995). "Tremor: is Parkinson’s disease a dynamical disease?" Chaos: an interdisciplinary. J. Nonlinear Sci. 5, 35–42. doi: 10.1063/1.166082 12780152

[ref8] Bostan A. C. Dum R. P. Strick P. L. (2010). The basal ganglia communicate with the cerebellum. Proc. Natl. Acad. Sci. U. S. A. 107, 8452–8456. doi: 10.1073/pnas.1000496107, PMID: 20404184 PMC2889518

[ref9] Brittain J.-S. Cagnan H. Mehta A. R. Saifee T. A. Edwards M. J. Brown P. (2015). Distinguishing the central drive to tremor in Parkinson's disease and essential tremor. J. Neurosci. 35, 795–806. doi: 10.1523/JNEUROSCI.3768-14.2015, PMID: 25589772 PMC4293424

[ref10] Brittain J.-S. Probert-Smith P. Aziz T. Z. Brown P. (2013). Tremor suppression by rhythmic transcranial current stimulation. Curr. Biol. 23, 436–440. doi: 10.1016/j.cub.2013.01.068, PMID: 23416101 PMC3629558

[ref11] DeLong M. R. Wichmann T. (2007). Circuits and circuit disorders of the basal ganglia. Arch. Neurol. 64, 20–24. doi: 10.1001/archneur.64.1.20 17210805

[ref12] Dirkx M. F. Zach H. Bloem B. R. Hallett M. Helmich R. C. (2018). The nature of postural tremor in Parkinson disease. Neurology 90, e1095–e1103. doi: 10.1212/WNL.0000000000005215, PMID: 29476038 PMC5880634

[ref13] Duval C. Daneault J.-F. Hutchison W. D. Sadikot A. F. (2016). A brain network model explaining tremor in Parkinson's disease. Neurobiol. Dis. 85, 49–59. doi: 10.1016/j.nbd.2015.10.009, PMID: 26459110

[ref14] Flanders M. (2009). “Voluntary movement” in Encyclopedia of neuroscience. eds. Binder M. D. Hirokawa N. Windhorst U. (Berlin, Heidelberg: Springer Berlin Heidelberg), 4371–4375.

[ref15] Fregni F. Boggio P. S. Santos M. C. Lima M. Vieira A. L. Rigonatti S. P.. (2006). Noninvasive cortical stimulation with transcranial direct current stimulation in Parkinson's disease. Mov. Disord. 21, 1693–1702. doi: 10.1002/mds.21012 16817194

[ref16] Fukumoto I. (1986). Computer simulation of parkinsonian tremor. J. Biomed. Eng. 8, 49–55. doi: 10.1016/0141-5425(86)90030-0, PMID: 3951209

[ref17] Gil L. M. Nunes T. P. Silva F. H. S. Faria A. C. D. Melo P. L. (2010). Analysis of human tremor in patients with Parkinson disease using entropy measures of signal complexity. Annu. Int. Conf. IEEE Eng. Med. Biol. Soc. 2010, 2786–2789. doi: 10.1109/IEMBS.2010.5626365 21095968

[ref18] Grabska-Barwińska A. Żygierewicz J. (2006). A model of event-related EEG synchronization changes in beta and gamma frequency bands. J. Theor. Biol. 238, 901–913. doi: 10.1016/j.jtbi.2005.07.001, PMID: 16099472

[ref19] Groth J. D. Sahin M. (2015). High frequency synchrony in the cerebellar cortex during goal directed movements. Front. Syst. Neurosci. 9:98. doi: 10.3389/fnsys.2015.00098 26257613 PMC4508504

[ref20] Haeri M. Sarbaz Y. Gharibzadeh S. (2005). Modeling the Parkinson’s tremor and its treatments. J. Theor. Biol. 236, 311–322. doi: 10.1016/j.jtbi.2005.03.014 15950988

[ref21] Hague S. M. Klaffke S. Bandmann O. (2005). Neurodegenerative disorders: Parkinson's disease and Huntington's disease. J. Neurol. Neurosurg. Psychiatry 76, 1058–1063. doi: 10.1136/jnnp.2004.060186, PMID: 16024878 PMC1739745

[ref22] Hari R. (2006). Action–perception connection and the cortical mu rhythm. Prog. Brain Res. 159, 253–260. doi: 10.1016/S0079-6123(06)59017-X 17071236

[ref23] Harikrishna A. Osei D. E. Kaminska M. W. Majumdar S. (2016). On numerical study of Parkinson tremor. bioRxiv. doi: 10.1101/065318

[ref24] Helmich R. C. Hallett M. Deuschl G. Toni I. Bloem B. R. (2012). Cerebral causes and consequences of parkinsonian resting tremor: a tale of two circuits? Brain 135, 3206–3226. doi: 10.1093/brain/aws023, PMID: 22382359 PMC3501966

[ref25] Hess C. W. (2013). Modulation of cortical-subcortical networks in Parkinson's disease by applied field effects. Front. Hum. Neurosci. 7:565. doi: 10.3389/fnhum.2013.00565 24062667 PMC3772338

[ref26] Hornykiewicz O. (2006). The discovery of dopamine deficiency in the parkinsonian brain. J. Neural Transm. 70, 9–15. doi: 10.1007/978-3-211-45295-0_3 17017502

[ref27] Hoshi E. Tremblay L. Féger J. Carras P. L. Strick P. L. (2005). The cerebellum communicates with the basal ganglia. Nat. Neurosci. 8, 1491–1493. doi: 10.1038/nn1544 16205719

[ref28] Hu Z. Hao M. Xu S. Xiao Q. Lan N. (2019). Evaluation of tremor interference with control of voluntary reaching movements in patients with Parkinson’s disease. J. Neuroeng. Rehabil. 16:38. doi: 10.1186/s12984-019-0505-0, PMID: 30866977 PMC6417201

[ref29] Kishore A. Meunier S. Popa T. (2014). Cerebellar influence on motor cortex plasticity: behavioral implications for Parkinson’s disease. Front. Neurol. 5:68. doi: 10.3389/fneur.2014.00068, PMID: 24834063 PMC4018542

[ref30] Lefaivre S. C. Brown M. J. N. Almeida Q. J. (2016). Cerebellar involvement in Parkinson’s disease resting tremor. Cerebellum Ataxias 3:13. doi: 10.1186/s40673-016-0051-5, PMID: 27280027 PMC4897799

[ref31] Lemon R. N. (2008). Descending pathways in motor control. Annu. Rev. Neurosci. 31, 195–218. doi: 10.1146/annurev.neuro.31.060407.125547 18558853

[ref32] Leondopulos S. Micheli-Tzanakou E (2004). A functional model based on single unit recordings from Parkinsonian brain. Proceedings of the 2004 IEEE International Conference onComputational Intelligence for Measurement Systems and Applications, 2004, Boston, MA, USA.

[ref33] Louis E. D. Machado D. G. (2015). Tremor-related quality of life: a comparison of essential tremor vs. Parkinson’s disease patients. Parkinsonism Relat. Disord. 21, 729–735. doi: 10.1016/j.parkreldis.2015.04.019, PMID: 25952960 PMC4764063

[ref34] Louis E. D. Tang M. X. Cote L. Alfaro B. Mejia H. Marder K. (1999). Progression of parkinsonian signs in Parkinson disease. Arch. Neurol. 56, 334–337. doi: 10.1001/archneur.56.3.334 10190824

[ref35] Marzban S. Ala T. S. Towhidkhah F. Forogh B. Habibi S. A. (2017). On the effects of transcranial direct current stimulation on hand movement in Parkinson’s disease: a primary study. Proceedings of the 2017 24th National and 2nd International Iranian Conference on Biomedical Engineering (ICBME), Tehran, Iran.

[ref36] MashhadiMalek M. Towhidkhah F. Gharibzadeh S. Daeichin V. Ali Ahmadi-Pajouh M. (2008). Are rigidity and tremor two sides of the same coin in Parkinson’s disease? Comput. Biol. Med. 38, 1133–1139. doi: 10.1016/j.compbiomed.2008.08.007, PMID: 18945424

[ref37] Morrison S. Newell K. M. (2000). Postural and resting tremor in the upper limb. Clin. Neurophysiol. 111, 651–663. doi: 10.1016/S1388-2457(99)00302-8 10727916

[ref38] Neuling T. Rach S. Herrmann C. (2013). Orchestrating neuronal networks: sustained after-effects of transcranial alternating current stimulation depend upon brain states. Front. Hum. Neurosci. 7:161. doi: 10.3389/fnhum.2013.00161, PMID: 23641206 PMC3639376

[ref39] Parker F. Tzourio N. Blond S. Petit H. Mazoyer B. (1992). Evidence for a common network of brain structures involved in Parkinsonian tremor and voluntary repetitive movement. Brain Res. 584, 11–17. doi: 10.1016/0006-8993(92)90872-7, PMID: 1515931

[ref40] Pincus S. M. Gladstone I. M. Ehrenkranz R. A. (1991). A regularity statistic for medical data analysis. J. Clin. Monit. 7, 335–345. doi: 10.1007/BF01619355 1744678

[ref41] Puschmann A. Wszolek Z. K. (2011). Diagnosis and treatment of common forms of tremor. Semin. Neurol. 31, 65–77. doi: 10.1055/s-0031-1271312, PMID: 21321834 PMC3907068

[ref42] Ramnani N. (2006). The primate cortico-cerebellar system: anatomy and function. Nat. Rev. Neurosci. 7, 511–522. doi: 10.1038/nrn1953, PMID: 16791141

[ref43] Stiefel K. M. Ermentrout G. B. (2016). Neurons as oscillators. J. Neurophysiol. 116, 2950–2960. doi: 10.1152/jn.00525.2015, PMID: 27683887 PMC5192043

[ref44] STMicroelectronics (2016). MEMS digital output motion sensor: ultra-low-power high-performance 3-axis "nano" accelerometer. LIS3DH.

[ref45] Strüber D. Rach S. Neuling T. Herrmann C. S. (2015). On the possible role of stimulation duration for after-effects of transcranial alternating current stimulation. Front. Cell. Neurosci. 9:311. doi: 10.3389/fncel.2015.00311, PMID: 26321912 PMC4530587

[ref46] Timmer J. Häussler S. Lauk M. Lücking C. H. (2000). "Pathological tremors: deterministic chaos or nonlinear stochastic oscillators?" Chaos: an interdisciplinary. J. Nonlinear Sci. 10, 278–288. doi: 10.1063/1.166494 12779383

[ref47] Timmermann L. Gross J. Dirks M. Volkmann J. Freund H. J. Schnitzler A. (2002). The cerebral oscillatory network of parkinsonian resting tremor. Brain 126, 199–212. doi: 10.1093/brain/awg022 12477707

[ref48] Watson Alberts W. (1972). A simple view of parkinsonian tremor. Electrical stimulation of cortex adjacent to the rolandic fissure in awake man. Brain Res. 44, 357–369. doi: 10.1016/0006-8993(72)90308-3 4561697

[ref49] Wong J. K. Cauraugh J. H. Ho K. W. D. Broderick M. Ramirez-Zamora A. Almeida L.. (2019). STN vs. GPi deep brain stimulation for tremor suppression in Parkinson disease: a systematic review and meta-analysis. Parkinsonism Relat. Disord. 58, 56–62. doi: 10.1016/j.parkreldis.2018.08.017, PMID: 30177491 PMC8980840

[ref50] Wu T. Hallett M. (2005). A functional MRI study of automatic movements in patients with Parkinson’s disease. Brain 128, 2250–2259. doi: 10.1093/brain/awh569 15958505

[ref51] Wu T. Hallett M. (2013). The cerebellum in Parkinson’s disease. Brain 136, 696–709. doi: 10.1093/brain/aws360, PMID: 23404337 PMC7273201

[ref52] Yu H. Sternad D. Corcos D. M. Vaillancourt D. E. (2007). Role of hyperactive cerebellum and motor cortex in Parkinson’s disease. Neuroimage 35, 222–233. doi: 10.1016/j.neuroimage.2006.11.047, PMID: 17223579 PMC1853309

